# A Quantitative and Comparative Study of Heroin-Related Metabolites in Different Postmortem Fluids and Tissues

**DOI:** 10.3390/toxics13030229

**Published:** 2025-03-20

**Authors:** Torki A. Zughaibi, Ziad Assiri, Ahmed Mirza, Hassan Alharbi, Abdulnasser E. Alzahrani, Sultan A. Alahmadi, Faiz Alsolami, Adel Al-Saadi, Mohamed Almoustady, Sultan Al-Zahrani, Majda Altowairqi, Ahmed I. Al-Asmari

**Affiliations:** 1 Department of Medical Laboratory Sciences, Faculty of Applied Medical Sciences, King Abdulaziz University, Jeddah 21589, Saudi Arabia; ziadahmdi@gmail.com (Z.A.); amirza1@kau.edu.sa (A.M.); 2 King Fahd Medical Research Center, King Abdulaziz University, Jeddah 21589, Saudi Arabia; malmistadi@kau.edu.sa; 3 Poison Control and Forensic Chemistry Center, Ministry of Health, Jeddah 21176, Saudi Arabia; dfhhh25@gmail.com (H.A.); abdulnassera@moh.gov.sa (A.E.A.); saalahamdi@moh.gov.sa (S.A.A.); fdalsolami@moh.gov.sa (F.A.); aaalsaadi@moh.gov.sa (A.A.-S.); suabalzahrani4@moh.gov.sa (S.A.-Z.); mmaltowairqi@moh.gov.sa (M.A.); 4 Special Toxicological Analysis Unit, Pathology and Laboratory Medicine DPLM, King Faisal Specialist Hospital and Research Center, Riyadh 11211, Saudi Arabia; 5 Faculty of Medicine, Alfaisal University, Riyadh 11533, Saudi Arabia

**Keywords:** LC-MS/MS, heroin, putrefaction, alternative samples, postmortem analysis and forensic toxicology

## Abstract

This study assessed and compared the postmortem concentrations of 6-monoacetylmorphine [6-MAM] and 6-acetylcodeine [6-AC], morphine, and codeine in various tissues and fluids from 52 postmortem cases related to heroin use. Samples were received at the Poison Control and Forensic Chemistry Center in Jeddah, Saudi Arabia, and analyzed using liquid chromatography–mass spectrometry. Descriptive and inferential statistics, including median, range, variability, and outliers, were used for analysis. The results showed significant variability in heroin and metabolite concentrations across different fluids and tissues. Tissue specimens were analyzed in 38 cases (73%), with 50% of cases exhibiting putrefaction. Blood and tissue samples were available in 39 cases, highlighting the need for alternative specimens in challenging cases. Notably, heroin metabolites were detected in unique matrices, such as nasal swabs, bladder tissues, lung tissues, and small intestine tissues, underscoring the potential of these samples in forensic investigations, especially when traditional bodily fluids are unavailable or compromised. These findings suggest that environmental factors, timing of substance use, and postmortem changes influence substance distribution, emphasizing the need to consider the location of death when interpreting toxicological results for accurate forensic analysis. This study provides valuable insights into the distribution, correlation, and significance of heroin and its metabolites in postmortem samples, aiding the confirmation of heroin overdose. These findings contribute to the limited data on postmortem cases in the Middle East and North Africa, particularly Saudi Arabia, supporting efforts to curb drug abuse in this region. This knowledge can inform public health strategies and forensic practices, ultimately aiding efforts to address and mitigate drug abuse.

## 1. Introduction

Heroin is a semi-synthetic opioid synthesized from morphine, a natural alkaloid extracted from the opium poppy that is used for recreational or medical purposes. It causes euphoria, happiness, and relaxation in its users as well as addiction and overdose. According to the World Health Organization (World, 2023) and the United Nations Office on Drug and Crime [1], heroin is responsible for most of the deaths that occur from opioid overdoses each year [2,3]. Heroin is converted to morphine during metabolism. Although codeine is not a direct metabolite, it may be detected following the administration of “street heroin” as a byproduct of heroin synthesis. This occurs when 6-acetylcodeine (6-AC) is formed and subsequently metabolized into codeine [4,5]. Morphine and codeine are natural alkaloids routinely prescribed for pain relief. These compounds and their metabolites, such as 6-monoacetylmorphine (6-MAM), which is a specific marker of heroin use, can be detected in postmortem samples [6,7]. Forensic toxicology faces several challenges, particularly with 6-MAM and 6-AC, which are known to have short half-lives and are often present in trace concentrations [5]. Therefore, it is crucial to use sensitive detection methods to identify these trace concentrations and provide valuable information for determining the source of the heroin used.

Heroin-related fatalities are a well-known cause of death and have been extensively studied for decades. However, it is important to recognize that scientific research is an ongoing process, and many issues still need to be explored and investigated. Existing data can become outdated or rely on older methodologies and techniques, which is particularly true in the case of heroin testing, especially for postmortem tissue distribution. For example, while the importance of vitreous humor in forensic analysis is well established, few studies have been conducted that considered the differences between ethnicities and genetic variations. This field of research seeks to gather information from around the globe to better understand causes of death and to introduce new specimens that may be available for forensic investigation, such as solid tissues and oral and nasal swabs. These specimens are crucial for determining the route of administration in some cases.

One key challenge is to determine the cause and manner of death in cases involving heroin [8,9,10]. A comprehensive analysis of postmortem samples, including blood, urine, bile, vitreous humor, and various other tissues, is deemed to be useful in both interpretating the cause of death and determining the levels of heroin and its metabolites. However, there is no agreement on the best methods and criteria for interpreting the postmortem results of heroin. Various studies have reported conflicting findings regarding the distribution, correlation, and significance of heroin and its metabolites in various postmortem samples. Therefore, more systematic and comprehensive studies are needed to provide reliable and accurate forensic information [8,9,10,11,12,13,14,15].

We analyzed the postmortem levels of morphine, codeine, 6-AC, and 6-MAM in various fluids and tissues from 52 cadavers related to opiate-related postmortem cases. In our previous investigation, we conducted the first epidemiological study of heroin-related deaths in Saudi Arabia by examining body fluid specimens over a period of 10 years. In the current study, we extended our analysis to include tissue specimen analysis and explore their used in heroin-related postmortem investigations. To the best of our knowledge, this is the first time heroin metabolites have been reported in postmortem cases involving lung, intestine, and bladder tissues. Additionally, we explored the use of nasal swabs as an alternative tool for detecting 6-MAM, 6-AC, morphine, and codeine and to identify the route of administration.

Our analysis focused on the variation and correlation of the concentrations of these substances with heroin use in different fluids and tissues. We employed a fully validated liquid chromatography–tandem mass spectrometry (LC-MS/MS) method using a quantitative and comparative approach. Statistical analyses were conducted to compare and correlate the levels of these drugs in various fluids and tissues.

The objective of this study was to provide the forensic community with insights into heroin-related postmortem cases and identify potential indicators that could be valuable in decomposition cases where limited samples are available for analysis. Data from postmortem cases are rarely reported in the Middle East and North Africa, especially in Saudi Arabia. It is widely accepted that heroin abuse in Saudi Arabia is uncommon. Consequently, most prevention, education, and intervention programs, as well as emergency department protocols, do not specifically address heroin abuse. This study is one of the few investigations to discuss the presence of heroin in the western area of Saudi Arabia. By enhancing knowledge and awareness, this study can help emergency departments and addiction centers to become more vigilant and better equipped to handle cases of heroin abuse.

## 2. Material and Methods

### 2.1. Materials

Opioids were used as analytes: 6-acetylmorphine hydrochloride (6-AM), morphine (MOR), 6-acetylcodeine (6-AC), and codeine monohydrate (COD). Analytes were purchased from Lipomed AG (Arlesheim, Switzerland) at a concentration of 1.00 mg/mL. Three deuterated opioids were used as the internal standards: 6-acetylemorphine-D3 hydrochloride trihydrate (6-AM-D3), morphine-D3 (MOR-D3), and codeine-D3 hydrochloride dihydrate (COD-D3). All the internal standards were purchased from Lipomed AG at a concentration of 1.00 mg/mL. High-performance liquid chromatography (HPLC)-grade solvents were used for opioid extraction and analysis. Methanol (99.8%), ethyl acetate, phosphate buffer (pH 6.0), hexane (ultrapure grade), acetic acid (0.1 M), dichloromethane (DCM), isopropanol (IPA), and ammonium formate were used as solvents, all of which were obtained from Sigma-Aldrich (Merck, KGaA), except for ammonium formate, which was purchased from Sigma-Aldrich (Saint Louis, MO, USA). The solvents were stored at room temperature (25 °C). Several consumables were used for sample preparation and analysis, including Clean Screen^®^ SPE (CSTHCU203, 200 mg/3 mL) cartridges, adjustable volume pipettes, disposable tips, sample vials, inserts with screw-top caps, Pyrex sample tubes (16 × 125 mL), disposable plastic transfer pipettes, syringes, and syringe filters. The cartridges were supplied by UCT Bristol (St. Louis, MO, USA), the pipettes and tips by Eppendorf AG (Hamburg, Germany), the vials, insert, and caps by Thermo Scientific (Langerwehe, Germany), the sample tubes by Neutrex^®^ (DWK Life Sciences, Wertheim, Germany), the transfer pipettes by S. G. H. (Lecca, Italy), and the syringes and filters by Ramy, Alshifa (Dammam, Saudi Arabia).

### 2.2. Instrumentation

In our previous studies [2,4], we analyzed 6-MAM, 6-AC, morphine, and codeine using two distinct LC-MS/MS systems, both of which were also employed in the current study. The initial system employed was a Thermo Finnigan LCQ Fleet ion trap, paired with a Surveyor LC system interface (Thermo Finnigan, San Jose, CA, USA), featuring ESI^+^ and SIM modes. Analyte separation was carried out using a Synergy Polar RP column, along with a matching guard column, both sourced from Phenomenex, Torrance, CA, USA. The column oven temperature was set to 30 °C and the autosampler tray was maintained at 4 °C. Ammonium formate buffer (10 mM, pH 3) was used as mobile phase A, whereas 100% acetonitrile served as mobile phase B, with a constant flow rate of 0.3 mL/min. The gradient ramp started with 3% mobile phase B for 3 min, increased to 15% B over the next 5 min, followed by 26% B over 7 min, reached 80% B for 13 min, and finally increased to 95% B over the last 2 min. The mobile phase was maintained for 3 min after 27 min of the analysis. Data were acquired using Xcalibur software 2.07 SP1 (Thermo Finnigan, San Jose, CA, USA).

Furthermore, the second analytical procedure was carried out using a Shimadzu LC-MS-8050 triple quadrupole mass spectrometer (Kyoto, Japan) with ESI^+^ and a Shimadzu Nexera UHPLC system, following a previously established method [16] to detect 6-MAM, 6-AC, morphine, and codeine. A Raptor Biophenyl column along with a corresponding guard column (Restek, USA) was used for analyte separation. The same mobile phase A and pure methanol as mobile phase B were used to maintain the previously mentioned flow rate. The gradient elution started with 3% B for 1 min, increased to 5% B over the next minute, and finally reached 95% B for 13 min. To re-equilibrate the column, mobile phase A was initially run for 1 min and held for an additional 4 min. MRM mode was employed to identify and quantify the analytes and internal standards based on their retention times and the detection of one or two product ions. Data acquisition and management were performed using LabSolution software (version 5.75; Shimadzu, Kyoto, Japan). The LC-MS/MS parameters for system A and B are listed in Table S1.

### 2.3. Sample Selection

All of the necessary approvals were obtained before the initiation of this study. Institutional Review Board approval was obtained from the Ministry of Health on 9 February 2020 (#H-01-J-002). The time between death and sample collection ranged from one to ten days, with less than 50% of cases showing signs of putrefaction. Each cadaver’s state of decomposition was assessed and categorized as non-putrefied, partially decomposed, or heavily decomposed, based on visual inspection and specific criteria. The present study followed a comparative analytical assay to quantify postmortem opiate/opioid concentrations collected from 52 cadavers (Table 1). The tissues included liver, kidney, lung, stomach, intestine, urinary bladder, and brain. Body fluids included blood, blood with sodium fluoride (BNaF), vitreous humor, urine, gastric contents, nasal secretions, bile, and pulmonary infiltrations from the chest. Blood samples were collected from subclavian sites in tubes containing 1% sodium fluoride. The sample collection details followed our previous study [2]. We also collected data from the Jeddah Poison Control Center (PCFCC) Forensic Toxicology Report database, encompassing all cases that tested positive for morphine, codeine, 6-AC, and 6-MAM between 2016 and 2021. In this study, we utilized previously established methods to analyze various fluid and tissue samples. Heroin and its metabolites were detected using two systems: the LC-MS/MS 8050 (Shimadzu Corporation, Japan) [11] and the Thermo Finnigan LCQ Fleet ion trap instrument with a Surveyor LC system interface (Thermo Finnigan, San Jose, CA, USA) [4], both equipped with electrospray ionization (ESI+) and selective reaction monitoring modes.

### 2.4. Sample Preparation

We diluted and homogenized 1 g of each solid tissue sample in a Stomacher bag for 5 min at a 2:1 *w*/*w* ratio (1% sodium fluoride solution/tissue) and 0.5 g of the homogenate was then transferred into separate 15 mL glass tubes. Subsequently, 50 μL of 6-MAM-D3, morphine-D3, and codeine-D3 as internal standard solution was added to each tube and mixed thoroughly. Finally, each tube was filled with 2 mL volume of 0.1 M phosphate buffer (pH 6).

#### Solid Phase Extraction

For the solid-phase extraction, a Clean Screen^®^ (CSDAU203) cartridge was activated sequentially with 2 mL of methanol, followed by 2 mL of deionized water, and then 2 mL of 0.1 M phosphate buffer (pH 6). The samples were centrifuged and then loaded onto the column, which was subsequently rinsed twice—first with 1 mL of deionized water, followed by 1 mL of 0.1 M acetic acid—and dried under full vacuum for 5 min. The column was subsequently rinsed twice with 1 mL of hexane, followed by the elution of fraction A using a 2 mL mixture of hexane and ethyl acetate (1:1, *v*/*v*). The elution tube was then removed. The column was then cleaned with 3 mL methanol and dried under vacuum for 2 min. Fraction B was eluted with 3 mL of a combination of solvents (dichloromethane/isopropanol/ammonium hydroxide, 78:20:2, *v*/*v*/*v*). Fractions (A and B) were evaporated using a nitrogen stream and subsequently reconstituted in 200 μL of the initial mobile phase. Finally, a 1 µL aliquot of this solution was analyzed by LC-MS/MS.

### 2.5. Method Validation

The method used in this investigation was validated in accordance with ANSI/ASB standards [17] and other established method validation protocols [18,19]. Two distinct LC-MS/MS methods were thoroughly validated using samples from bodily fluids and tissues before their application in this study. Previous publications have documented complete method validation for blood and other specimens [11,12]. For calibration and quality control, negative human postmortem samples confirmed to be free of drugs were used. Over the course of the 10-year study period, the method parameters were periodically reoptimized and revalidated as part of ongoing quality control and updates to policies and procedures mandated by the Jeddah Poison Control Center, Jeddah, Saudi Arabia (Table 2).

6-MAM and 6-AC are not stable, which leads to a decrease in the concentration of these analytes in improperly stored specimens. Therefore, a sensitive method is required for measuring these metabolites. The stability of these heroin metabolites was investigated in an autosampler using three controls that were previously used in precision studies. The samples were re-analyzed after 24 h, 48 h, and one week.

The most important feature of such analytical methods is the investigation of the limit of detection (LOD) in the proposed matrix of interest and the workable lower limit of quantification (LOQ) for quantifying unstable analytes at very low concentrations (≤1 ng/mL). Elevated morphine concentrations in urine and bile were used as the upper limit of quantification (ULOQ) and assessed for all heroin-related analytes. The sensitivity analysis was performed in accordance with the ANSI/ASB method validation guidelines [17]. Negative blank samples from different matrices were included in each batch of samples, without any standards or internal standards. Negative blank samples with internal standards were only used to investigate method selectivity, and negative blank samples were run following the calibrator with the highest concentration to investigate any carryover.

Matrix effects were investigated using post-extraction addition, as reported by Matuszewski et al. [19]. Six different autopsy specimens were analyzed for each matrix of interest using the optimized method. These matrices were negative, and three concentrations (low, medium, and high) were analyzed (each concentration was repeated five times). The same approach was used to calculate extraction recoveries for these different matrix sources, while standards were added before extraction and internal standards were added post-extraction for the recovery experiment. Matrix effects were obtained by comparison with neat standards prepared in the initial mobile phase, and the extraction recovery value was assessed via comparison with the matrix effect results, as described by Matuszewski et al. [19].

### 2.6. Statistical Analysis

Data analysis was performed using the Statistical Package for Social Sciences (IBM, SPSS, version 25), including the Kruskal–Wallis test. Preliminary descriptive statistics indicated that drug concentrations in various fluids and tissues were significantly right-skewed, with median values generally lower than arithmetic means. As a result, the distributions were best represented by medians and interquartile ranges rather than means.

## 3. Results

The validated analysis method was acceptable (Table 2), and the sensitivity of the proposed method for detecting incredibly low concentrations of 6-MAM, 6-AC, morphine, and codeine and higher concentrations of morphine in urine and bile had an LOQ of 1 ng/mL for all analytes.

**Figure 1:**
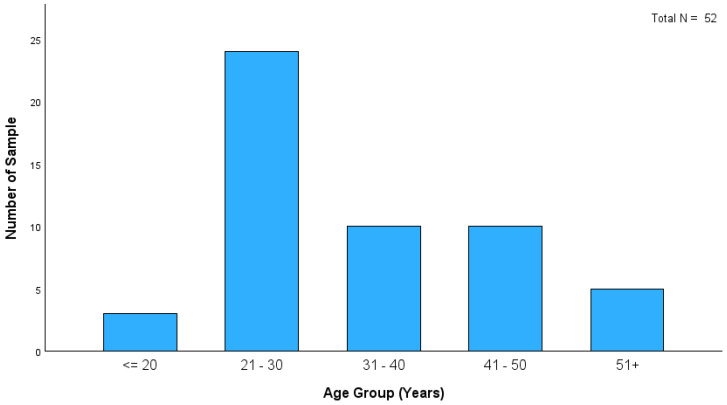
Distribution of deceased individuals by age group.

**Figure 2:**
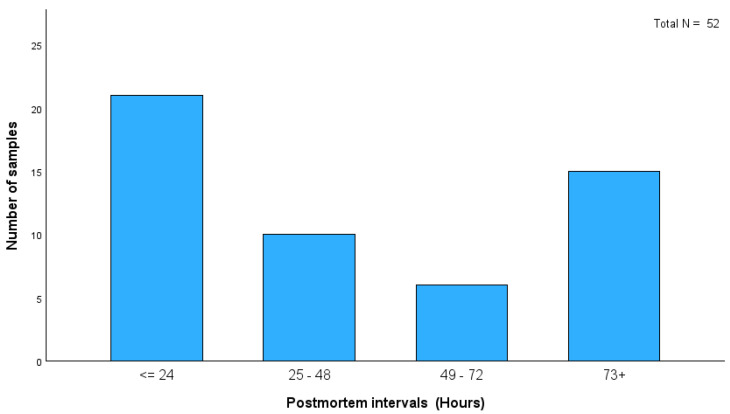
Distribution of postmortem intervals in hours.

We analyzed samples from 52 cadavers for morphine, codeine, 6-AC, and 6-MAM (Table 3). Figure 1 shows that the age distribution of heroin-related deaths in this study indicates that most cases were within the 21–30 age group. This finding highlights the significant prevalence of heroin-related fatalities among young adults. Although the distribution of PMIs among heroin-related deaths in this study showed that most cases fell within the ≤ 24 h category (21 cases), longer PMIs were observed in the remaining cases (Figure 2). This led to an increased incidence of putrefaction in almost 50% of cases, limiting the availability of biofluid specimens (Table 4). Consequently, solid tissue analysis has been used in some putrefied cases, particularly those found outdoors.

Fifty-two postmortem cases were included in this study and analyzed for morphine, codeine, 6-AC, and 6-MAM (Table 1). Tissue specimens were submitted for analysis in 38 cases (73%). These tissue specimens were helpful for interpretation, as 50% of the cases exhibited putrefaction (19 cases partially and 6 cases heavily; Table 4). Blood and BNaF samples were available in 39 cases, making the interpretation of heroin-related fatalities challenging without alternative and complementary specimens. The 6-MAM was quantified in 22 blood samples and 10 BNaF samples out of the 52 cases. Urine and vitreous humor were missing in most samples, but were positive for 6-MAM in 33 and 34 cases, respectively. The distribution of 6-MAM across the various body fluids and tissue specimens is presented in Table 3. 6-AC is often excluded from routine postmortem cases because of its trace levels and short half-life. Using a sensitive LC-MS/MS method efficiently detected 6-AC, and in this study, 6-MAM was detected in parallel with 6-AC in some biofluid and tissue samples. Gastric contents were found to be an ideal matrix for detecting 6-AC (Table 3), while most biofluids showed positive cases, but at trace concentrations, often lower than 6-MAM. Notably, 6-AC was not detected in cases without 6-MAM.

In the current study, tissue samples were very effective in identifying the source of opioids by detecting 6-MAM and 6-AC in cases in which no blood, urine, or vitreous humor samples were available (Table 1). 6-MAM was detected in four out of eight brain tissue samples, eleven out of sixteen stomach wall tissue samples, four out of five small intestine tissue samples, three out of seven lung tissue samples, and three out of three bladder tissue samples. However, these tissues were less effective in detecting 6-AC, with only a few samples testing positive: one brain tissue, four stomach wall tissues, and three out of four small intestine tissues, whereas none of the bladder and lung tissues tested positive for 6-AC.

Nasal swabs collected during autopsy were analyzed in this study. Four of the five nasal swabs showed high concentrations of 6-MAM, indicating that heroin was sniffed in these cases. Heroin could be detected in these cases; however, this method was not designed for heroin quantification. These swabs showed no 6-AC, and the morphine concentrations were often lower than those of 6-MAM. In one case, only morphine was detected, suggesting that nasal swabs could be used to identify the route of administration. However, further research is required to validate this hypothesis due to the limited number of reported cases.

Morphine concentration spanned across various postmortem samples, including both fluids and tissues (Table 3). The bile sample exhibited the highest median morphine concentration among all fluids and tissues for all comparisons with other fluids, whereas the vitreous humor had the lowest median concentration for all comparisons with other fluids. Notably, fluids, such as urine and bile, displayed extreme outliers in morphine levels, whereas the blood and vitreous humor had the smallest ranges. The chart indicates that the liver and bladder had the highest median morphine levels for all comparisons with other tissues, whereas the brain had the lowest median morphine concentrations for all comparisons with other tissues. The brain also demonstrated greater variability in morphine levels than other tissues.

The codeine levels varied significantly across fluids and tissues. As shown in Table 3, urine had the highest median codeine level among all fluids, whereas vitreous humor and BNaF had the lowest. The bile and gastric contents exhibited outliers in codeine levels. Additionally, Table 3 indicates that most tissue samples showed no significant differences in codeine concentrations.

Based on the correlation analysis between tissues and body fluids, several significant relationships were identified with the significance threshold set at 0.05. A strong positive correlation was observed between morphine and codeine in bile (r = 0.774, *p* < 0.05), indicating a significant relationship. Additionally, a moderate positive correlation was found between 6-MAM and codeine in urine (r = 0.321, *p* < 0.05), suggesting their co-occurrence in this fluid. In vitreous humor, the correlation between morphine and 6-MAM was also significant (r = 0.271, *p* < 0.05), highlighting their potential relationship.

Furthermore, the correlation between morphine and codeine in blood was strong and significant (r = 0.587, *p* < 0.05), emphasizing the importance of analyzing these substances together. In tissue samples, significant positive correlations were observed between morphine and 6-MAM in the liver (r = 0.452, *p* < 0.05), and between codeine and 6-AC in the kidney (r = 0.369, *p* < 0.05). These findings illustrate that the distribution of 6-MAM, 6-AC, morphine, and codeine across different tissues and body fluids can provide comprehensive insights for forensic analyses. A strong correlation was noted between morphine and codeine in the small intestine (r = 0.510, *p* < 0.05), and between 6-MAM and morphine in the stomach wall (r = 0.432, *p* < 0.05). The bladder showed that a significant correlation was observed between morphine and 6-MAM (r = 0.398, *p* < 0.05), whereas the lung demonstrated a positive correlation between morphine and codeine (r = 0.344, *p* < 0.05). In the brain, a notable correlation was observed between 6-AC and morphine (r = 0.278, *p* < 0.05).

These findings illustrate that the distribution of 6-MAM, 6-AC, morphine, and codeine across different tissues and body fluids can provide comprehensive insights for forensic analyses. Higher concentrations of morphine were found in tissues than in BNaF, with elevated levels of these substances in bile and urine and lower levels in the vitreous humor. The significant correlations and distribution patterns underscore the importance of analyzing various postmortem matrices to accurately determine the presence of heroin metabolites and other opioids. This comprehensive analysis enhances the accuracy of forensic investigations by providing valuable insights into the distribution and relationship of substances across different biological samples, thus reinforcing the benefits of this study.

Table S2 differentiates between heroin-only detected drugs (29 cases) and polydrug detection (19 cases), including substances, such as methamphetamine, ethanol, cocaine, and their metabolites. Some heroin-only cases were positive for amphetamine and cannabinoids, although these drugs are not often associated with death. Methamphetamine was the most frequently detected drug in 13 cases of polydrug intoxication, followed by cannabinoids, amphetamines, alprazolam, cocaine, and pregabalin. The latter was considered a metabolite of methamphetamine; in seven cases, amphetamine was detected without methamphetamine. Amphetamine is the most commonly used drug in this region.

Analysis of the causes of death revealed that these cases could be classified as monointoxication (Table S2). Of the 52 cases, 29 (55.8%) were attributed solely to heroin, 19 (36.5%) were classified as heroin-related deaths, and 4 (7.7%) were undetermined. This breakdown highlights the prevalence of heroin-only fatalities and emphasizes the significance of heroin as a primary cause of death in these instances.

A study on the modes of death revealed that most heroin-related fatalities were classified as accidental (Table S3). Among these, various biofluids and tissues showed significant levels of morphine, codeine, 6-AC, and 6-MAM, providing valuable insights for forensic analysis. There were comparatively few homicidal and unknown cases, with notable outliers in certain fluids and tissues. As 50% of the deceased were putrefied, it was challenging to assign the mode of death or to determine the time since the last heroin intake. In some cases, evidence such as heroin bags, syringes, and nasal swabs was used. Putrefaction makes it difficult to identify the injection site when the body begins to decompose.

The distribution of substances across various locations of death (Table S4), such as cars, homes, hospitals, hotel rooms, and outdoors, showed significant variability. Higher concentrations of morphine and 6-MAM were found in homes and outdoor settings, whereas codeine was consistently detected at all locations. Bile samples demonstrate elevated morphine levels, especially in outdoor environments. Urine samples showed notably higher 6-MAM and morphine concentrations in homes, with substantial variations among outdoor locations. Lung samples showed consistent detection of morphine and codeine, with higher concentrations in the outdoor samples. The small intestine and stomach wall samples displayed varied levels of substances, with the notable detection of morphine and codeine in homes and outdoors. Bladder samples revealed the presence of 6-MAM and morphine in a few cases, predominantly at home. These findings suggest that environmental factors, timing of substance use, and postmortem changes influence substance distribution, emphasizing the need to consider the location of death when interpreting toxicological results for accurate forensic analysis.

## 4. Discussion

The results of this study have important implications for forensic toxicology and investigation of heroin-related deaths. This study was unique because it analyzed multiple samples from the same deceased individual and included a significant number of cases. Notably, heroin metabolite levels varied among the specimens and remained detectable in fluids for a limited time. They were also traceable in the blood, urine, bile, and vitreous humor [5,12]. The urine and gastric contents exhibited higher concentrations of heroin metabolites, whereas detection in other specimens, such as the liver and kidney, was infrequent [9,10,11,20]. Challenges were encountered regarding the availability of urine samples, or, in some cases, deaths occurred immediately before metabolite release into the urine. The detection of 6-MAM is not solely reliant on sampling; several other factors must be considered. It is unstable both in vivo and in vitro, which necessitates proper sample storage. In certain cases, weather conditions are important and may lead to discrepancies between specimens [2,21,22]. Negative results in the blood, which is the gold standard for cause-of-death interpretation, can occur because of these factors. The detection of 6-MAM and 6-AC in the urine or vitreous humor may not always correlate with death, as their release from the vitreous humor may be delayed, and they may not be present in the urine before death or missing due to short elapsed time between intake or administration. Other factors, such as evidence surrounding the deaths (presence of heroin) and the occasional presence of a syringe attached to the deceased body, may play a role [2]. The present study improves the interpretation of heroin-related deaths by showing how various conditions affect the detection and concentration of 6-MAM, 6-AC, morphine, and codeine in multiple postmortem samples even when the body decomposes.

In this study, we report four cases in which 6-MAM was detected and one case in which 6-AC was detected in nasal swabs. These specimens can be used as alternative indicators to determine whether death was recent, whether heroin was the opioid used, and to indicate the route of administration. Furthermore, in three cases, 6-MAM, morphine, and codeine were detected in bladder tissues, suggesting that, although few cases were reported in this study, these matrices are promising. Rodda et al. [23] highlight the importance of bladder wash in providing the reliable detection of drugs and their metabolites, including heroin metabolites, even in putrefied cases where other biofluids are compromised. This finding underscores the value of bladder tissue as a crucial tool in forensic analysis, particularly in challenging postmortem scenarios. The results from the current investigation are consistent with Rodda et al.’s findings and provide additional insights into the detection of 6-MAM, morphine, and codeine.

Recent work also highlights the importance of stomach wall tissues for the detection of 6-MAM, 6-AC, morphine, and codeine [13]. Eleven out of sixteen cases were positive for 6-MAM and morphine in stomach wall tissues, while 6-AC was detected in four cases. Additionally, this study confirms the usefulness of intestinal tissues, especially in cases where no bodily fluid samples are available or in cases of delayed death where no 6-MAM is detected in blood samples. Four out of five small intestine cases were positive for 6-MAM, and three were positive for 6-AC. To the best of our knowledge, this is the first report of the detection of 6-MAM, 6-AC, morphine, and codeine in nasal swabs, the small intestine, and the bladder in postmortem cases.

Moreover, in heroin-related fatalities, only one study has reported heroin metabolites in lung tissue. Moriya and Hashimoto [24] documented a heroin- and methamphetamine-related fatality in which only morphine was detected following heroin injection, with almost equal levels of free and total morphine (1140 ng/g and 2270 ng/g, respectively). In the current study, morphine and codeine were detected in all lung tissues, with median concentrations of 350 and 30 ng/g, respectively. Interestingly, we found 6-MAM positive in three cases with concentrations of 5 ng/g, 4 ng/g, and 25 ng/g, while no 6-AC was detected. To the best of our knowledge, this is the first report of 6-MAM and codeine detection in lung tissue. The low detectability of 6-MAM can be attributed to the long postmortem intervals (PMIs) and susceptibility of the lung tissues to degradation. We believe that these findings, along with the detailed analysis and discussion presented in our manuscript, add value to the existing literature and provide a foundation for future research.

Al-Asmari [13] conducted a correlation analysis of heroin-related metabolites in various postmortem samples from 15 deceased individuals. Their analysis revealed that most samples exhibited strong and significant correlations between morphine and codeine, whereas 6-MAM had a weak or no correlation in most samples. They also reported the maximum and minimum levels of these metabolites in different tissues and fluids. Although the results of the present study showed some similarities to Al-Asmari’s report, there were some differences, such as the number of samples, types of samples, median levels, outliers, and contrast between different tissues and fluids.

Maskell et al. [8] examined the correlation between free morphine in femoral blood and other biological samples. They reported that cardiac blood, bone marrow, muscle, and brain showed significant correlations with femoral blood, whereas liver and vitreous humor were not correlated. The results of the present study shared some similarities with Maskell’s report, such as higher levels of morphine in fluids compared with that in tissues, higher morphine levels in bile and urine, and lower morphine levels in the vitreous humor.

Thaulow et al. [10] investigated the ratios of morphine and its metabolites across various postmortem matrices to determine their relationship to the rapidity of death. Their findings revealed that the morphine-3-glucuronide-to-morphine and morphine-6-glucuronide-to-morphine ratios were significantly lower in cases of rapid death than in delayed death in most fluids but not in tissues. Additionally, they observed that the morphine ratio in the vitreous humor relative to the peripheral blood was notably lower in the rapid-death group. They also reported morphine/codeine ratios across different matrices, distinguishing between the cases of heroin and codeine consumption.

The present study found higher concentrations of morphine in tissues than in BNaF, elevated levels of morphine in bile and urine, and lower levels in the vitreous humor and blood samples. The high morphine concentrations in tissues can be interpreted as resulting from the deconjugation of morphine glucuronides in vivo with increasing postmortem intervals (PMIs) [11,24]. Previous studies have shown significant variability in morphine concentrations across different body fluids and tissues. For example, Moriya and Hashimoto [24] found large amounts of total morphine in the stomach contents, liver, and lung, with variability in the proportion of free morphine. This aligns with our findings that bile exhibits the highest median concentration and variability, whereas vitreous humor shows the lowest concentration and minimal variability. The anatomical structure of vitreous humor contributes to its slow release and high stability of 6-MAM, while bile acts as a storage depot for opioids [5,15,25]. The presence of a heroin biomarker in this study was the best tool for identifying opioid sources.

It is well known that morphine is cleared from the plasma as a morphine glucuronide in urine. However, in patients who chronically use heroin, morphine can persist in bodily fluids and tissue specimens for an extended period, which complicates the interpretation. This persistence is due to reabsorption from the kidneys through a process called enterohepatic recirculation or repeated use of heroin to reach euphoria [26]. It has been found that solid tissues such as the liver and kidneys rarely detect 6-MAM, whereas other tissues such as the stomach wall, lungs, and brain are more suitable for its detection. This can be attributed to the pH of these tissues, such as the stomach and its contents, or due to enterohepatic recirculation [13]. This supports the observation that blood and blood BNaF have similar median concentrations with narrow interquartile ranges, indicating less variability compared to other fluids, such as bile and urine. The higher concentrations of morphine in bile can be attributed to enterohepatic circulation, where morphine is reabsorbed from the intestines and returned to the liver, leading to higher accumulation in bile [12,27]. This phenomenon is well documented in the literature and explains the elevated morphine levels observed in the bile samples. Overall, our findings are consistent with the existing literature on the distribution of morphine in different body fluids and tissues [8,10,28,29]. The significant differences in morphine concentrations among the fluid types, as suggested by the Kruskal–Wallis test, highlight the importance of considering the specific characteristics of each fluid when interpreting toxicological results.

Liver tissue can be opposite to that of kidney tissue in cases of type of heroin metabolites, while morphine glucuronide can be degraded a few days following deaths in liver and kidney samples [11,24,28]; on the other hand, they are appropriate for analyzing free morphine but not morphine conjugates because the conjugates are quickly excreted into urine. While free morphine is stable in liver and kidney samples at temperatures from 4.0 to 37.0 °C, the morphine conjugates turn into free morphine at temperatures from 18 to 37 °C in less than 10 days [24]. The kidney has a higher affinity for free morphine than conjugated morphine. In contrast, most morphine found in the liver is conjugated. The time span between death and analysis can cause some of the conjugated morphine in the liver to be degraded into its free form. This was supported by a previous study [13], where it was found that free morphine in the liver degradation was four times greater than that in blood, which can be explained by postmortem redistribution and/or morphine conjugate degradation to free morphine in liver specimens. Therefore, free morphine is more logically detected in cases of decomposition.

This study provides valuable insights into the distribution of heroin metabolites in various postmortem tissues and fluids, particularly within the context of the Middle East and North Africa (MENA) region. Our findings highlight the importance of considering regional differences in drug abuse patterns and the need for tailored forensic practices. The detection of heroin metabolites in unique matrices, such as nasal swabs, bladder tissues, and small intestine tissues, underscores the potential of these samples in forensic investigations, especially in cases where traditional bodily fluids are unavailable or compromised.

The use of advanced LC-MS/MS systems and validated methods ensured the robustness and reliability of our results, thereby offering a reference for future studies. Our research also emphasizes the practical implications for forensic practitioners, particularly in estimating the postmortem interval and confirming heroin administration. The variability in metabolite concentrations across different tissues can provide valuable information for forensic investigations, aiding in the interpretation of cases and the identification of the route of administration.

Furthermore, our study addresses the need for updated methodologies and techniques in heroin testing, as the existing literature may be based on outdated technologies. By contributing new data and perspectives, we hope to inform and enhance forensic practices, ultimately improving the accuracy and effectiveness of forensic investigations in the MENA region and beyond.

The present study has some limitations that may compromise the validity and applicability of the results. First, there were missing samples, such as femoral blood, that could have provided more reliable results. The potential bias in interpreting morphine levels due to the absence of femoral blood has been thoroughly addressed, highlighting its reliability in postmortem toxicology and the possible inaccuracies from other blood sources [21,30]. Second, the unavailability of hair samples during this study, which are vital complementary specimens for interpreting addiction status, represents another limitation. Hair samples are especially useful when traditional bodily fluids are unavailable or compromised as they can provide insights into the history of heroin use. Another limitation is the scarcity of epidemiological studies in the region and the rarity of reports on heroin-related fatalities [31]. These limitations may affect the accuracy, precision, and representativeness of the results, and limit the generalizability of the findings to other populations or settings. Future studies should use longer periods and more diverse samples, collect more data during the postmortem interval, and consider other factors that may influence the results, including more samples that may reflect the chronic or acute use of heroin, and incorporate more postmortem results from different cities across the Kingdom of Saudi Arabia.

In conclusion, this study not only adds to the existing body of knowledge, but also opens up new avenues for research and application in forensic science. We believe that our findings will serve as a foundation for future studies and contribute to the ongoing efforts to address and mitigate the impact of heroin abuse.

## 5. Conclusions

This study provides key insights into the distribution of heroin metabolites in postmortem tissues and fluids within the MENA region, emphasizing the value of novel matrices like nasal swabs, bladder, small intestine, and lung tissues in forensic investigations. These findings underscore the practical implications for estimating postmortem intervals, confirming heroin administration, and enhancing forensic methodologies. By contributing new data and perspectives, this study lays the groundwork for future research and advances forensic practices in the MENA region and beyond.

## Figures and Tables

**Table 1:** Summary of calibration and quality control methods for LC-MS/MS analysis.

Parameter	Method
Calibration Curves	Prepared for each new batch according to sample type
Linear Dynamic Range (LDR)	10-point calibration curve: 1000, 500, 250, 100, 50, 25, 10, 5, 1, 0.5 ng/mL for fluid samples and 1000, 500, 250, 100, 50, 25, 10, 5, 1, 0.5 ng/g for tissue samples.
Positive Quality Control (QC) Standards	Established at 25 ng/mL, 100 ng/mL, and 800 ng/mL for fluid samples and at 25 ng/g, 100 ng/g, and 800 ng/g for tissue samples.
Precision Analysis	Analyzed on the same day, repeated over five consecutive days (five replicates per concentration)

**Table 2:** Summary of key method validation parameters and outcomes.

Parameter	Details
Sensitivity	LOQ of 1 ng/mL for fluid samples and 1 ng/g for tissue samples for all analytes
Autosampler Temperature	Set to 4 °C during stability experiments
Stability of Controls	Stable up to one week, concentrations within ±10% of target
Polydrug Distinction	Method distinguished between analytes of interest and co-ingested drugs
Chromatogram Peaks	No peaks observed following injection of blank samples, internal standards, or analyte standards
Linearity	Coefficients of determination > 0.99 for 6-MAM, 6-AC, morphine, and codeine
LOD Values	Estimated from ten calibration curves, ranged between 0.2 and 0.4 ng/mL for fluid samples and between 0.2 and 0.4 ng/g for tissue samples.
LOQ Evaluation	Spiked with 1 ng/mL of each analyte, results from freshly prepared calibration curves
Precision, Accuracy, Stability	Within 15% of nominal value for precision, accuracy, dilution, and autosampler stability
Carryover Contamination	No contamination detected from previous positive control tests

**Table 3:** Postmortem concentrations of 6-acetylmorphine, 6-cetylcodeine, morphine, and codeine in various tissues and fluids.

Samples	Metabolites	Number of Samples	Concentrations		
			Median	Minimum	Maximum
Blood without preservative (ng/mL)	6-Mononoacetylmorphine	10	7	n.d.	17
	6-Acetylcodeine	2	n.d.	n.d.	n.d.
	Morphine	34	107	13	1927
	Codeine	31	12	2	94
Blood with sodium fluoride preservative (ng/mL)	6-Mononoacetylmorphine	22	10	1	251
	6-Acetylcodeine	5	3	2	12
	Morphine	39	83	4	1222
	Codeine	32	13	1	76
Urine (ng/mL)	6-Mononoacetylmorphine	33	335	1	18,876
	6-Acetylcodeine	13	8	n.d.	201
	Morphine	35	1238	23	42,264
	Codeine	34	135	n.d.	12,110
Vitreous humor (ng/mL)	6-Mononoacetylmorphine	34	22	3	243
	6-Acetylcodeine	7	3	n.d.	14
	Morphine	39	48	3	891
	Codeine	35	14	1	143
Gastric contents (ng/mL)	6-Mononoacetylmorphine	26	161	17	3802
	6-Acetylcodeine	20	62	5	540
	Morphine	32	243	25	1515
	Codeine	29	39	3	1325
Liver (ng/g)	6-Mononoacetylmorphine	2	1.60	1.20	2.00
	6-Acetylcodeine	n.d.	n.d.	n.d.	n.d.
	Morphine	34	150	2	1030
	Codeine	23	10	10	100
Kidneys (ng/g)	6-Mononoacetylmorphine	2	n.d.	n.d.	n.d.
	6-Acetylcodeine	n.d.	n.d.	n.d.	n.d.
	Morphine	27	300	40	4500
	Codeine	23	24	3	100
Bile (ng/mL)	6-Mononoacetylmorphine	17	14	2	360
	6-Acetylcodeine	3	4	3	10
	Morphine	31	2200	133	41,100
	Codeine	28	40	1	227
Stomach wall tissue (ng/g)	6-Mononoacetylmorphine	11	60	4	485
	6-Acetylcodeine	4	29	2	320
	Morphine	16	120	5	1300
	Codeine	14	30	5	260
Brain (ng/g)	6-Mononoacetylmorphine	4	25	10	309
	6-Acetylcodeine	1	3	3	3
	Morphine	8	100	35	1750
	Codeine	7	14	3	40
Bladder (ng/g)	6-Mononoacetylmorphine	3	33	13	289
	6-Acetylcodeine	n.d.	n.d.	n.d.	n.d.
	Morphine	3	350	154	1903
	Codeine	3	19	8	45
Small intestine tissue (ng/g)	6-Mononoacetylmorphine	4	73	10	415
	6-Acetylcodeine	3	6	3	185
	Morphine	5	150	135	1290
	Codeine	5	10	10	70
Lung tissue (ng/g)	6-Mononoacetylmorphine	3	5	4	25
	6-Acetylcodeine	n.d.	n.d.	n.d.	n.d.
	Morphine	7	310	130	450
	Codeine	7	30	8	55
Nasal swab (ng/swab)	6-Mononoacetylmorphine	4	439	158	2100
	6-Acetylcodeine	1	8	8	8
	Morphine	5	51	17	337
	Codeine	5	11	4	23

n.d.: not applicable or not detected.

**Table 4:** Distribution of 6-acetylmorphine, 6-cetylcodeine, morphine, and codeine in non-putrefied, partially putrefied, and heavily putrefied samples.

Samples Type	Putrefaction Status	Non-Putrefied				Partially Putrefied				Heavily Putrefied			
	Number of Cases	27				19				6			
	Metabolites	Number of Samples	Concentrations			Number of Samples	Concentrations			Number of Samples	Concentrations		
			Median	Minimum	Maximum		Median	Minimum	Maximum		Median	Minimum	Maximum
Blood without preservative (ng/mL)	6-Mononoacetylmorphine	7	8	1	17	4	1	0	17	N.S.	n.a.	n.a.	n.a.
	6-Acetylcodeine	2	n.a.	n.a.	n.a.	3	0	0	2	N.S.	n.a.	n.a.	n.a.
	Morphine	16	104	13	1125	16	95	32	1927	2	792	715	869
	Codeine	13	18	2	94	16	10	3	63	2	45	44	46
Blood with sodium fluoride preservative (ng/mL)	6-Mononoacetylmorphine	13	12	0	43	7	3	0	251	2	14	10	17
	6-Acetylcodeine	3	2	n.d.	9	5	2	0	12	N.S.	n.a.	n.a.	n.a.
	Morphine	22	100	4	827	15	70	13	1222	2	162	10	313
	Codeine	17	19	1	76	14	8	2	39	1	24	24	24
Urine (ng/mL)	6-Mononoacetylmorphine	18	425	1	4557	15	312	5	18,876	1	58	58	58
	6-Acetylcodeine	7	8	n.d.	201	6	11	1	162	N.S.	.	.	.
	Morphine	18	971	23	20,424	16	1131	72	42,264	1	1457	1457	1457
	Codeine	17	128	n.d.	972	16	137	3	12,110	1	605	605	605
Vitreous humor (ng/mL)	6-Mononoacetylmorphine	19	37	7	243	15	21	3	72	N.S.	n.a.	n.a.	n.a.
	6-Acetylcodeine	4	3	n.d.	14	3	3	3	6	0	n.a.	n.a.	n.a.
	Morphine	22	55	5	891	16	44	3	162	1	15	15	15
	Codeine	20	16	1	143	15	12	3	31	N.S.	n.a.	n.a.	n.a.
Gastric contents (ng/mL)	6-Mononoacetylmorphine	14	566	30	3802	10	97	17	661	2	111	84	138
	6-Acetylcodeine	11	97	5	540	9	58	20	376	N.S.	n.a.	n.a.	n.a.
	Morphine	17	239	25	1515	13	224	29	481	2	589	247	931
	Codeine	14	40	3	1325	13	32	12	92	2	28	16	39
Liver (ng/g)	6-Mononoacetylmorphine	2	1.60	1.20	2.00	N.S.	n.a.	n.a.	n.a.	N.S.	n.a.	n.a.	n.a.
	6-Acetylcodeine	N.S.	n.a.	n.a.	n.a.	N.S.	n.a.	n.a.	n.a.	N.S.	n.a.	n.a.	n.a.
	Morphine	17	85.00	15.00	810.00	12	170.00	22.00	2100.00	5	650.00	22.00	1031.00
	Codeine	10	9.50	1.00	50.00	10	10.50	3.00	30.00	3	18.00	17.00	28.00
Kidneys (ng/g)	6-Mononoacetylmorphine	N.S.	n.a.	n.a.	n.a.	1	1.00	1.00	1.00	1	4.00	4.00	4.00
	6-Acetylcodeine	N.S.	n.a.	n.a.	n.a.	N.S.	n.a.	n.a.	n.a.	N.S.	n.a.	n.a.	n.a.
	Morphine	14	225.00	40.00	1458.00	10	316.00	110.00	558.00	3	598.00	535.00	3485.00
	Codeine	11	25.00	3.00	70.00	9	20.00	8.00	30.00	3	28.00	21.00	95.00
Bile (ng/mL)	6-Mononoacetylmorphine	7	41.000	9.000	160.000	8	13.000	2.000	354.000	2	52.500	2.000	103.000
	6-Acetylcodeine	N.S.	n.a.	n.a.	n.a.	3	4.000	3.000	5.000	N.S.	n.a.	n.a.	n.a.
	Morphine	17	2348.000	173.000	41,100.000	10	1750.500	133.000	7700.000	4	1920.500	292.000	40,383.000
	Codeine	15	40.000	3.000	227.000	10	24.500	1.300	120.000	3	13.000	5.000	189.000
Stomach wall tissue (ng/g)	6-Mononoacetylmorphine	6	23	4	485	4	125	15	329	1	170	170	170
	6-Acetylcodeine	2	2	2	2	2	188	55	320	N.S.	n.a.	n.a.	n.a.
	Morphine	8	118	11	1100	6	61	5	1300	2	285	160	410
	Codeine	6	28	6	260	6	27	5	70	2	n.a.	n.a.	n.a.
Brain (ng/g)	6-Mononoacetylmorphine	N.S.	n.a.	n.a.	n.a.	3	15	10	35	1	n.a.	n.a.	n.a.
	6-Acetylcodeine	N.S.	n.a.	n.a.	n.a.	1	3	3	3	N.S.	n.a.	n.a.	n.a.
	Morphine	2	53	35	70	4	78	40	125	2	923	95	1750
	Codeine	1	5	5	5	4	18	3	40	2	16	14	17
Bladder (ng/g)	6-Mononoacetylmorphine	N.S.	n.a.	n.a.	n.a.	1	289	289	289	2	23	13	33
	6-Acetylcodeine	N.S.	n.a.	n.a.	n.a.	N.S.	n.a.	n.a.	n.a.	N.S.	n.a.	n.a.	n.a.
	Morphine	N.S.	n.a.	n.a.	n.a.	1	1903	1903	1903	2	252	154	350
	Codeine	N.S.	n.a.	n.a.	n.a.	1	19	19	19	2	27	8	45
Small intestine tissue (ng/g)	6-Mononoacetylmorphine	1	n.a.	n.a.	n.a.	3	13	10	415	N.S.	n.a.	n.a.	n.a.
	6-Acetylcodeine	N.S.	n.a.	n.a.	n.a.	3	6	3	185	N.S.	n.a.	n.a.	n.a.
	Morphine	2	143	135	150	3	295	135	1290	N.S.	n.a.	n.a.	n.a.
	Codeine	2	28	10	45	3	10	10	70	N.S.	n.a.	n.a.	n.a.
Lung tissue (ng/g)	6-Mononoacetylmorphine	2	15	5	25	1	n.a.	n.a.	n.a.	N.S.	n.a.	n.a.	n.a.
	6-Acetylcodeine	N.S.	n.a.	n.a.	n.a.	N.S.	n.a.	n.a.	n.a.	N.S.	n.a.	n.a.	n.a.
	Morphine	3	310	233	310	3	350	130	410	1	n.a.	n.a.	n.a.
	Codeine	3	30	20	33	3	35	10	55	1	n.a.	n.a.	n.a.
Nasal swab (ng/swab)	6-Mononoacetylmorphine	3	163	158	2100	N.S.	n.a.	n.a.	n.a.	1	n.a.	n.a.	n.a.
	6-Acetylcodeine	1	n.a.	n.a.	n.a.	N.S.	n.a.	n.a.	n.a.	N.S.	n.a.	n.a.	n.a.
	Morphine	3	168	51	337	1	n.a.	n.a.	n.a.	1	n.a.	n.a.	n.a.
	Codeine	3	11	10	23	1	n.a.	n.a.	n.a.	1	n.a.	n.a.	n.a.

n.d.: not applicable or not detected, N.S.: no sample available.

## Data Availability

Whole data are included in the manuscript and will be available online.

## Supplementary Materials

The following supporting information can be downloaded at: https://www.mdpi.com/article/10.3390/toxics13030229/s1, Table S1: Liquid chromatography electrospray ionization tandem mass spectrometry (LC-MS/MS); Table S2: Cause of deaths and concentration of 6-MAM, 6-AC, morphine, and codeine across various postmortem samples; Table S3: Mode of death and concentration of 6-MAM, 6-AC, morphine, and codeine in various postmortem samples; Table S4: Location of death and concentration of 6-MAM, 6-AC, morphine, and codeine in various postmortem samples.

## List of Abbreviations

MENA	Middle East and North Africa
6-MAM	6-monoacetylmorphine
