# Supplementary material for: A Quantitative and Comparative Study of Heroin-Related Metabolites in Different Postmortem Fluids and Tissues
Source: Toxics. 2025 Mar 20;13(3):229. doi: 10.3390/toxics13030229 (PMC11945869; doi:10.3390/toxics13030229)
Supplement: Supplementary file 1 [file toxics-13-00229-s001.zip › toxics-3430891-supplementary.pdf]

Table S1 Liquid chromatography electrospray ionization tandem mass spectrometry (LC-MS/MS)

Analytes	Internal Standards	LCQ Fleet			LC-MS 8050		
		**Retention time. (min)**	**Quantifier Ion**	**Qualifier Ion**	**Retention time. (min)**	**Quantifier Ion**	**Qualifier Ion**
6-Mononoacetylmorphine	6-Mononoacetylmorphine-D3	15.1	m/z = 328→211	m/z = 328→268	6.9	m/z = 328→165	m/z = 328→221
6-Mononoacetylmorphine-D3	-	15.1	m/z = 331→165	m/z = 331→221	7	m/z = 331→165	m/z = 331→221
6-Acetylcodeine	Codeine-d3	19	m/z = 342→225	m/z = 342→282	9.3	m/z = 342→225	m/z = 342→165
Morphine	Morphine-d3	8.4	m/z = 286→201	m/z = 286→229	4.7	m/z = 286→165	m/z = 286→153
Morphine-d3	-	8.2	m/z = 289→201	m/z = 289→229	4.6	m/z = 289→165	m/z = 289→153
Codeine	Codeine-d3	13.9	m/z = 300→215	m/z = 300→243	6.8	m/z = 300→165	m/z = 300→44
Codeine-d3	-	13.9	m/z = 303→215	m/z = 303→243	6.7	m/z = 303→165	m/z = 303→199

Table S2 Cause of Deaths and Concentration of 6-Mononoacetylmorphine, 6-Acetlcodeine, morphine and codeine Across Various Postmortem Samples

Samples	Unit		Cause of deaths											
			Heroin only				Heroin related deaths				Undetermined			
		Number of cases	29				19				4			
				Concentrations				Concentrations				Concentrations		
		Heroin metabolites	N	Median	Minimum	Maximum	N	Median	Minimum	Maximum	N	Median	Minimum	Maximum
Blood without preservative	ng/mL	6-Mononoacetylmorphine	5	8	1	17	5	3	1	17	n.s.	n.s.	n.s.	n.s.
		6-Acetylcodeine	1	n.a.	n.a.	n.a.	2	n.a.	n.a.	n.a.	n.s.	n.s.	n.s.	n.s.
		Morphine	21	121	15	1927	12	102	13	1125	1	n.a.	n.a.	n.a.
		Codeine	21	11	2	63	10	13	3	94	n.s.	n.s.	n.s.	n.s.
Blood with Sodium Fluoride preservative	ng/mL	6-Mononoacetylmorphine	11	12	0	251	10	10	1	43	n.s.	n.s.	n.s.	n.s.
		6-Acetylcodeine	3	9	2	12	2	3	2	3	n.s.	n.s.	n.s.	n.s.
		Morphine	22	69	4	1222	16	100	9	827	1	10	10	10
		Codeine	19	12	1	34	13	19	3	76	n.s.	n.s.	n.s.	n.s.
Urine	ng/mL	6-Mononoacetylmorphine	18	339	20	18876	16	335	1	4557	n.s.	n.s.	n.s.	n.s.
		6-Acetylcodeine	6	62	1	201	7	2	0	23	n.s.	n.s.	n.s.	n.s.
		Morphine	19	1325	23	42264	16	696	45	20424	n.s.	n.s.	n.s.	n.s.
		Codeine	18	187	0	12110	16	118	3	972	n.s.	n.s.	n.s.	n.s.
Vitreous humor	ng/mL	6-Mononoacetylmorphine	19	21	3	243	15	35	5	133	n.s.	n.s.	n.s.	n.s.
		6-Acetylcodeine	2	10	5	14	5	3	0	6	n.s.	n.s.	n.s.	n.s.
		Morphine	20	48	3	162	17	55	5	891	2	n.a.	n.a.	n.a.
		Codeine	20	11	3	39	15	16	1	143	n.s.	n.s.	n.s.	n.s.
Gastric Contents		6-Mononoacetylmorphine	14	179	17	3600	12	147	22	3802	n.s.	n.s.	n.s.	n.s.
		6-Acetylcodeine	12	62	7	540	8	74	5	446	n.s.	n.s.	n.s.	n.s.
		Morphine	19	184	29	885	12	326	34	1515	1	n.a.	n.a.	n.a.
		Codeine	18	41	3	232	11	32	19	1325	n.s.	n.s.	n.s.	n.s.
Liver	ng/mL	6-Mononoacetylmorphine	2	1.60	1.20	2.00	n.s.	n.s.	n.s.	n.s.	C	n.s.	n.s.	n.s.
		6-Acetylcodeine	n.s.	n.s.	n.s.	n.s.	n.s.	n.s.	n.s.	n.s.	n.s.	n.s.	n.s.	n.s.
		Morphine	20	112.50	15.00	810.00	11	180.00	22.00	2100.00	3	230.00	22.00	310.00
		Codeine	12	11.50	5.00	50.00	11	11.00	1.00	37.00	n.s.	n.s.	n.s.	n.s.
Kidneys	ng/g	6-Mononoacetylmorphine	n.s.	n.s.	n.s.	n.s.	2	n.a.	n.a.	n.a.	n.s.	n.s.	n.s.	n.s.
		6-Acetylcodeine	n.s.	n.s.	n.s.	n.s.	0	.	.	.	n.s.	n.s.	n.s.	n.s.
		Morphine	16	277.50	40.00	3485.00	9	340.00	140.00	598.00	2	n.a.	n.a.	n.a.
		Codeine	12	22.00	3.00	95.00	9	28.00	4.00	45.00	2	26.00	25.00	27.00

Bile	ng/g	6-Mononoacetylmorphine	9	13.000	2.000	354.000	7	14.000	2.000	70.000	1	n.a.	n.a.	n.a.
		6-Acetylcodeine	1	n.a.	n.a.	n.a.	2	n.a.	n.a.	n.a.	0	.	.	.
		Morphine	18	2364.000	133.000	41100.000	10	2088.500	300.000	9808.000	3	292.000	173.000	6000.000
		Codeine	17	37.000	1.300	227.000	10	36.500	3.000	120.000	1	n.a.	n.a.	n.a.
Stomach-Wall Tissue	ng/mL	6-Mononoacetylmorphine	5	35	4	329	6	125	10	485	n.s.	n.s.	n.s.	n.s.
		6-Acetylcodeine	1	n.a.	n.a.	n.a.	3	55	2	320	n.s.	n.s.	n.s.	n.s.
		Morphine	9	29	5	410	6	195	55	1300	1	n.a.	n.a.	n.a.
		Codeine	8	21	5	55	5	35	5	260	1	n.a.	n.a.	n.a.
Brain		6-Mononoacetylmorphine	1	n.a.	n.a.	n.a.	3	15	10	35	n.s.	n.s.	n.s.	n.s.
		6-Acetylcodeine	0	.	.	.	1	n.a.	n.a.	n.a.	n.s.	n.s.	n.s.	n.s.
		Morphine	4	83	35	1750	4	78	40	125	n.s.	n.s.	n.s.	n.s.
		Codeine	3	14	5	17	4	18	3	40	n.s.	n.s.	n.s.	n.s.
Bladder	ng/g	6-Mononoacetylmorphine	1	n.a.	n.a.	n.a.	1	13	13	13	1	33	33	33
		6-Acetylcodeine	n.s.	n.s.	n.s.	n.s.	n.s.	n.s.	n.s.	n.s.	n.s.	n.s.	n.s.	n.s.
		Morphine	1	n.a.	n.a.	n.a.	1	n.a.	n.a.	n.a.	1	n.a.	n.a.	n.a.
		Codeine	1	n.a.	n.a.	n.a.	1	n.a.	n.a.	n.a.	1	n.a.	n.a.	n.a.
Small-Intestine Tissue	ng/g	6-Mononoacetylmorphine	n.s.	n.s.	n.s.	n.s.	3	13	10	415	1	n.a.	n.a.	n.a.
		6-Acetylcodeine	n.s.	n.s.	n.s.	n.s.	3	6	3	185	n.s.	n.s.	n.s.	n.s.
		Morphine	1	150	150	150	3	295	135	1290	1	n.a.	n.a.	n.a.
		Codeine	1	45	45	45	3	10	10	70	1	n.a.	n.a.	n.a.
Lung Tissue	ng/g	6-Mononoacetylmorphine	1	5	5	5	2	15	4	25	n.s.	n.s.	n.s.	n.s.
		6-Acetylcodeine	0	.	.	.	0	.	.	.	n.s.	n.s.	n.s.	n.s.
		Morphine	2	380	310	450	4	292	130	410	1	n.a.	n.a.	n.a.
		Codeine	2	21	8	33	4	28	10	55	1	n.a.	n.a.	n.a.
Nasal Swab	ng/swab	6-Mononoacetylmorphine	2	437	158	715	2	1132	163	2100	n.s.	n.s.	n.s.	n.s.
		6-Acetylcodeine	0	.	.	.	1	8	8	8	n.s.	n.s.	n.s.	n.s.
		Morphine	3	44	17	168	2	194	51	337	n.s.	n.s.	n.s.	n.s.
		Codeine	3	10	4	17	2	17	11	23	n.s.	n.s.	n.s.	n.s.

N: No. of Samples.
n.s. No sample
n.d.: Not detected
n.a. Not available

Table S3 Mode of Death and Concentration of 6-Mononoacetylmorphine, 6-Acetlcodeine, morphine and codeine in Various Postmortem Samples.

Samples	Unit	Mode of Death	Accidental				Homicidal				Unknown			
		Number of Cases	45				3				4			
				Concentrations				Concentrations				Concentrations		
		Heroin Metabolites	N	Median	Minimum	Maximum	N	Median	Minimum	Maximum	N	Median	Minimum	Maximum
Blood without preservative	ng/mL	6-Mononoacetylmorphine	11	7	1	17	n.s.	n.s.	n.s.	n.s.	n.s.	n.s.	n.s.	n.s.
		6-Acetylcodeine	1	n.a.	n.a.	n.a.	n.s.	n.s.	n.s.	n.s.	n.s.	n.s.	n.s.	n.s.
		Morphine	31	105	13	1927	3	150	23	715	n.s.	n.s.	n.s.	n.s.
		Codeine	29	12	2	94	2	n.a.	n.a.	n.a.	n.s.	n.s.	n.s.	n.s.
Blood with Sodium Fluoride preservative	ng/mL	6-Mononoacetylmorphine	20	10	0	251	1	n.a.	n.a.	n.a.	1	n.a.	n.a.	n.a.
		6-Acetylcodeine	8	2	.n.d.	12	n.s.	n.s.	n.s.	n.s.	n.s.	n.s.	n.s.	n.s.
		Morphine	36	88	4	1222	1	n.a.	n.a.	n.a.	2	172	10	334
		Codeine	30	13	1	76	1	n.a.	n.a.	n.a.	1	n.a.	n.a.	n.a.
Urine	ng/mL	6-Mononoacetylmorphine	32	305	1	18876	1	482	482	482	1	n.a.	n.a.	2830
		6-Acetylcodeine	13	8	n.d	201	n.s.	n.s.	n.s.	n.s.	n.s.	n.s.	n.s.	n.s.
		Morphine	33	1023	23	42264	1	2185	2185	2185	1	2840	2840	2840
		Codeine	33	128	n.d	12110	n.s.	n.s.	n.s.	n.s.	1	340	340	340
Vitreous humor	ng/mL	6-Mononoacetylmorphine	32	22	3	243	1	n.a.	n.a.	n.a.	1	80	80	80
		6-Acetylcodeine	7	3	n.d	14	n.s.	n.s.	n.s.	n.s.	n.s.	n.s.	n.s.	n.s.
		Morphine	35	50	3	891	2	n.a.	n.a.	n.a.	2	38	15	60
		Codeine	33	14	1	143	1	n.a.	n.a.	n.a.	1	n.a.	n.a.	n.a.
Gastric Contents	ng/mL	6-Mononoacetylmorphine	26	161	17	3802	n.s.	n.s.	n.s.	n.s.	n.s.	n.s.	n.s.	n.s.
		6-Acetylcodeine	20	62	5	540	n.s.	n.s.	n.s.	n.s.	n.s.	n.s.	n.s.	n.s.
		Morphine	30	243	29	1515	2	454	25	882	n.s.	n.s.	n.s.	n.s.
		Codeine	28	38	3	1325	1	57	57	57	n.s.	n.s.	n.s.	n.s.
Liver	ng/g	6-Mononoacetylmorphine	2	n.a.	n.a.	n.a.	n.s.	n.s.	n.s.	n.s.	n.s.	n.s.	n.s.	n.s.
		6-Acetylcodeine	n.s.	n.s.	n.s.	n.s.	n.s.	n.s.	n.s.	n.s.	n.s.	n.s.	n.s.	n.s.
		Morphine	30	163.50	15.00	2100.00	2	223.50	52.00	395.00	2	126.00	22.00	230.00
		Codeine	22	10.50	1.00	50.00	1	18.00	18.00	18.00	n.s.	n.s.	n.s.	n.s.
Kidneys	ng/mL	6-Mononoacetylmorphine	2	2.50	1.00	4.00	n.s.	n.s.	n.s.	n.s.	n.s.	n.s.	n.s.	n.s.
		6-Acetylcodeine	n.s.	n.s.	n.s.	n.s.	n.s.	n.s.	n.s.	n.s.	n.s.	n.s.	n.s.	n.s.

		Morphine	24	277.50	40.00	1400.00	2	2471.50	1458.00	3485.00	1	270.00	270.00	270.00
		Codeine	20	22.00	3.00	70.00	2	59.50	24.00	95.00	1	25.00	25.00	25.00
Bile	ng/mL	6-Mononoacetylmorphine	13	13.000	2.000	354.000	2	72.000	41.000	103.000	2	81.000	2.000	160.000
		6-Acetylcodeine	3	4.000	3.000	5.000	n.s.	n.s.	n.s.	n.s.	n.s.	n.s.	n.s.	n.s.
		Morphine	25	1829.000	133.000	41100.000	3	9438.000	173.000	40383.000	3	2577.000	292.000	6000.000
		Codeine	24	36.500	1.300	165.000	2	208.000	189.000	227.000	2	11.500	10.000	13.000
Stomach-Wall Tissue	ng/g	6-Mononoacetylmorphine	11	60	4	485	n.s.	n.s.	n.s.	n.s.	n.s.	n.s.	n.s.	n.s.
		6-Acetylcodeine	4	29	2	320	n.s.	n.s.	n.s.	n.s.	n.s.	n.s.	n.s.	n.s.
		Morphine	14	76	5	1300	1	410	410	410	1	230	230	230
		Codeine	12	21	5	260	1	55	55	55	1	45	45	45
Brain	ng/g	6-Mononoacetylmorphine	4	25	10	309	n.s.	n.s.	n.s.	n.s.	n.s.	n.s.	n.s.	n.s.
		6-Acetylcodeine	1	n.a.	n.a.	n.a.	n.s.	n.s.	n.s.	n.s.	n.s.	n.s.	n.s.	n.s.
		Morphine	6	70	35	125	2	910	70	1750	n.s.	n.s.	n.s.	n.s.
		Codeine	6	12	3	40	1	17	17	17	n.s.	n.s.	n.s.	n.s.
Bladder	ng/g	6-Mononoacetylmorphine	2	151	13	289	n.s.	n.s.	n.s.	n.s.	1	33	33	33
		6-Acetylcodeine	0	.	.	.	n.s.	n.s.	n.s.	n.s.	0	.	.	.
		Morphine	2	1029	154	1903	n.s.	n.s.	n.s.	n.s.	1	350	350	350
		Codeine	2	n.a.	n.a.	n.a.	n.s.	n.s.	n.s.	n.s.	1	45	45	45
Small-Intestine Tissue	ng/g	6-Mononoacetylmorphine	3	13	10	415	n.s.	n.s.	n.s.	n.s.	1	132	132	132
		6-Acetylcodeine	3	6	3	185	n.s.	n.s.	n.s.	n.s.	0	.	.	.
		Morphine	3	295	135	1290	1	150	150	150	1	135	135	135
		Codeine	3	10	10	70	1	45	45	45	1	10	10	10
Lung Tissue	ng/g	6-Mononoacetylmorphine	3	5	4	25	n.s.	n.s.	n.s.	n.s.	n.s.	n.s.	n.s.	n.s.
		6-Acetylcodeine	0	.	.	.	n.s.	n.s.	n.s.	n.s.	n.s.	n.s.	n.s.	n.s.
		Morphine	5	310	130	410	1	450	450	450	1	310	310	310
		Codeine	5	33	10	55	1	8	8	8	1	30	30	30
Nasal Swab	ng/swab	6-Mononoacetylmorphine	1	163	163	163	2	437	158	715	1	2100	2100	2100
		6-Acetylcodeine	1	n.a.	n.a.	n.a.	n.s.	n.s.	n.s.	n.s.	n.s.	n.s.	n.s.	n.s.
		Morphine	2	177	17	337	2	106	44	168	1	51	51	51
		Codeine	2	14	4	23	2	14	10	17	1	11	11	11

N: Number of samples
n.s. No sample
n.d.: Not detected
n.a. Not available

Table S4 Location of Death and Concentration of 6-Mononoacetylmorphine, 6-Acetlcodeine, morphine and codeine and Metabolites in Various Postmortem Samples.

Samples	Unit	Location	Car			Home			Hospital			Hotel Room			Outdoor		
		No. of Cases	8			21			2			2			19		
				Concentrations			Concentrations			Concentrations			Concentrations			Concentrations	
		Heroin Metabolites	N	Median	Range	N	Median	Range	N	Median	Range	N	Median	Range	N	Median	Range
Blood without preservative	ng/mL	6-Mononoacetylmorphine	2	6	3-10	4	5	1-20	n.s.	n.s.	n.s.	n.s.	n.s.	n.s.	4	10	1-17
		6-Acetylcodeine	1	n.a.	n.a.	1	n.a.	n.a.	n.s.	n.s.	n.s.	n.s.	n.s.	n.s.	1	n.a.	n.a.
		Morphine	5	105	50-235	14	93	13-1927	1	n.a.	n.a.	2	n.a.	n.a.	12	151	32-1125
		Codeine	5	11	3-24	13	12	2-63	n.s.	n.s.	n.s.	1	n.a.	n.a.	12	17	3-94
Blood with Sodium Fluoride preservative	ng/mL	6-Mononoacetylmorphine	4	14	3-24	9	10	1-251	1	n.a.	n.a.	n.s	n.s	n.s	7	12	3-43
		6-Acetylcodeine	1	n.a.	n.a.	2	n.a.	n.a.	n.s.	n.s.	n.s.	n.s	n.s	n.s	2	n.a.	n.a.
		Morphine	6	88	50-210	17	102	4-1222	1	n.a.	n.a.	1	n.a.	n.a.	14	64	10-827
		Codeine	6	20	3-34	14	11	1-54	n.s.	n.s.	n.s.	n.s	n.s	n.s	12	14	2-76
Urine	ng/mL	6-Mononoacetylmorphine	6	21	1-701	18	328	5-18876	n.s.	n.s.	n.s.	1	n.a.	n.a.	9	426	54-2830
		6-Acetylcodeine	3	1	n.d.-2	6	12	n.d.-201	n.s.	n.s.	n.s.	n.s	n.s	n.s	4	62	1-162
		Morphine	6	91	50-1023	18	971	70-42264	n.s.	n.s.	n.s.	2	n.a.	n.a.	9	2185	20-9279
		Codeine	6	9	3-107	18	160	5-12110	n.s.	n.s.	n.s.	2	n.a.	n.a.	8	405	n.d.-602
Vitreous humor	ng/mL	6-Mononoacetylmorphine	7	18	5-91	14	29	10-243	n.s.	n.s.	n.s.	n.s	n.s	n.s	13	24	3-133
		6-Acetylcodeine	2	n.a.	n.a.	4	5	3-14	n.s.	n.s.	n.s.	n.s	n.s	n.s	1	n.a.	n.a.
		Morphine	7	37	5-254	15	55	3-487	1	n.a.	n.a.	2	n.a.	n.a.	14	54	10-891
		Codeine	6	21	1-124	15	14	5-67	n.s.	n.s.	n.s.	1	n.a.	n.a.	13	12	3-143
Gastric Contents	ng/mL	6-Mononoacetylmorphine	6	112	20-3802	15	138	30-1597	n.s.	n.s.	n.s.	n.s	n.s	n.s	5	193	20-3592
		6-Acetylcodeine	4	20	5-446	11	58	10-376	n.s.	n.s.	n.s.	n.s	n.s	n.s	5	97	40-540
		Morphine	7	263	38-1515	17	223	30-931	1	25	25	1	n.a.	n.a.	6	582	30-885
		Codeine	7	37	20-1325	16	31	3-240	n.s.	n.s.	n.s.	1	n.a.	n.a.	5	73	40-176
Liver		6-Mononoacetylmorphine	n.s.	n.s.	n.s.	1	n.a.	n.a.	1	n.a.	n.a.	n.s	n.s	n.s	n.s	n.s	n.s
		6-Acetylcodeine	n.s.	n.s.	n.s.	0	.	.	n.s.	n.s.	n.s.	n.s	n.s	n.s	n.s	n.s	n.s

	ng/g	Morphine	5	140	30-2100	13	167.00	15-1031	1	n.a.	n.a.	n.s	n.s	n.s	15	200	22-810
		Codeine	4	12	1-20	11	10.00	5-50	n.s.	n.s.	n.s.	n.s	n.s	n.s	8	13	3-50
Kidneys	ng/mL	6-Mononoacetylmorphine	n.s.	n.s.	n.s.	2	n.a.	n.a.	n.s.	n.s.	n.s.	n.s	n.s	n.s	n.s	n.s	n.s
		6-Acetylcodeine	n.s.	n.s.	n.s.	n.s.	n.s.	n.s.	n.s.	n.s.	n.s.	n.s	n.s	n.s	n.s	n.s	n.s
		Morphine	4	160.00	367.00	12	280	40-765	n.s.	n.s.	n.s.	n.s	n.s	n.s	11	325	130-3-485
		Codeine	3	10.00	45.00	12	22	3-52	n.s.	n.s.	n.s.	n.s	n.s	n.s	8	25	20-95
Bile	ng/mL	6-Mononoacetylmorphine	1	n.a.	n.a.	10	14	2-354	n.s.	n.s.	n.s.	n.s	n.s	n.s	6	72	2-160
		6-Acetylcodeine	1	n.a.	n.a.	n.a.	n.a.	n.a.	n.s.	n.s.	n.s.	n.s	n.s	n.s	1	n.a.	n.a.
		Morphine	4	7150	300-41100	15	885	133-7700	1	n.a.	n.a.	1	1350.000	1350.000	10	4925	300-41081
		Codeine	4	60	3-165	15	24	1-120	n.s.	n.s.	n.s.	1	8.000	8.000	8	63	10-227
Stomach-Wall Tissue	ng/g	6-Mononoacetylmorphine	1	n.a.	n.a.	8	125	6-485	n.s.	n.s.	n.s.	n.s	n.s	n.s	2	n.a.	n.a.
		6-Acetylcodeine	1	n.a.	n.a.	3	55	2-320	n.s.	n.s.	n.s.	n.s	n.s	n.s	n.s	n.s	n.s
		Morphine	2	76	85	9	55	5-1300	n.s.	n.s.	n.s.	n.s	n.s	n.s	5	230	15-410
		Codeine	2	14	18	8	22	5-260	n.s.	n.s.	n.s.	n.s	n.s	n.s	4	45	24-55
Brain	ng/g	6-Mononoacetylmorphine	n.s.	n.s.	n.s.	3	15	10-35	n.s.	n.s.	n.s.	n.s	n.s	n.s	1	n.a.	n.a.
		6-Acetylcodeine	n.s.	n.s.	n.s.	1	n.a.	n.a.	n.s.	n.s.	n.s.	n.s	n.s	n.s	n.s	n.s	n.s
		Morphine	1	n.a.	n.a.	4	78	40-125	n.s.	n.s.	n.s.	n.s	n.s	n.s	3	95	70-1750
		Codeine	1	n.a.	n.a.	4	18	5-40	n.s.	n.s.	n.s.	n.s	n.s	n.s	2	n.a.	n.a.
Bladder	ng/g	6-Mononoacetylmorphine	n.s.	n.s.	n.s.	2	151	10-289	n.s.	n.s.	n.s.	n.s	n.s	n.s	1	n.a.	n.a.
		6-Acetylcodeine	n.s.	n.s.	n.s.	n.s.	n.s.	n.s.	n.s.	n.s.	n.s.	n.s	n.s	n.s	n.s	n.s	n.s
		Morphine	n.s.	n.s.	n.s.	2	1029	1903	n.s.	n.s.	n.s.	n.s	n.s	n.s	1	n.a.	n.a.
		Codeine	n.s.	n.s.	n.s.	2	n.a.	n.a.	n.s.	n.s.	n.s.	n.s	n.s	n.s	1	n.a.	n.a.
Small-Intestine Tissue	ng/g	6-Mononoacetylmorphine	n.s.	n.s.	n.s.	3	13	10-415	n.s.	n.s.	n.s.	n.s	n.s	n.s	1	n.a.	n.a.
		6-Acetylcodeine	n.s.	n.s.	n.s.	3	6	3-185	n.s.	n.s.	n.s.	n.s	n.s	n.s	n.s	n.s	n.s
		Morphine	n.s.	n.s.	n.s.	3	295	140-1290	n.s.	n.s.	n.s.	n.s	n.s	n.s	2	n.a.	n.a.
		Codeine	n.s.	n.s.	n.s.	3	10	1070	n.s.	n.s.	n.s.	n.s	n.s	n.s	2	n.a.	n.a.
Lung Tissue	ng/g	6-Mononoacetylmorphine	n.s.	n.s.	n.s.	2	n.a.	n.a.	n.s.	n.s.	n.s.	n.s	n.s	n.s	1	n.a.	n.a.
		6-Acetylcodeine	n.s.	n.s.	n.s.	n.s.	n.s.	n.s.	n.s.	n.s.	n.s.	n.s	n.s	n.s	n.s	n.s	n.s
		Morphine	n.s.	n.s.	n.s.	4	292	130-410	n.s.	n.s.	n.s.	n.s	n.s	n.s	3	310	310-450
		Codeine	n.s.	n.s.	n.s.	4	28	10-55	n.s.	n.s.	n.s.	n.s	n.s	n.s	3	30	8-33
Nasal Swab	ng/swab	6-Mononoacetylmorphine	n.s.	n.s.	n.s.	n.s.	n.s.	n.s.	n.s.	n.s.	n.s.	1	163	163	3	715	2100
		6-Acetylcodeine	n.s.	n.s.	n.s.	n.s.	n.s.	n.s.	n.s.	n.s.	n.s.	1	8	8	n.s	n.s	n.s
		Morphine	n.s.	n.s.	n.s.	1	17	17	0	.	.	1	337	337	3	51	168
		Codeine	n.s.	n.s.	n.s.	1	4	4	0	.	.	1	23	23	3	11	17

N: Number of samples
n.s. No sample

n.d.: Not detected
n.a. Not available
